# Viral Metagenomics in Patients Who Underwent Allogeneic Hematopoietic Stem Cell Transplantation (HSCT): A Brazilian Experience

**DOI:** 10.3390/microorganisms12122557

**Published:** 2024-12-12

**Authors:** Gabriel Montenegro de Campos, Thalita Cristina de Mello Costa, Roberta Maraninchi Silveira, Ian Nunes Valença, Rafael dos Santos Bezerra, Luiz Guilherme Darrigo Junior, Ana Carolina de Jesus Vieira, Camila Campos Mesquita, Patrícia da Silva Laurindo, Renato Guerino Cunha, Simone Kashima, Dimas Tadeu Covas, Belinda Pinto Simões, Sandra Coccuzzo Sampaio, Maria Carolina Elias, Marta Giovanetti, Svetoslav Nanev Slavov

**Affiliations:** 1 Blood Center of Ribeirão Preto, Ribeirão Preto Medical School, University of São Paulo, Ribeirão Preto 05508-220, SP, Brazil; gabrielmdecampos@usp.br (G.M.d.C.); robsilveira.maraninchi@gmail.com (R.M.S.); nunes006@usp.br (I.N.V.); rafaelbezerra50@yahoo.com (R.d.S.B.); skashima@hemocentro.fmrp.usp.br (S.K.); 2 Department of Medical Imaging, Hematology and Oncology, Ribeirão Preto Medical School, University of São Paulo, Ribeirão Preto 14040-900, SP, Brazil; tcmcosta@hcrp.usp.br (T.C.d.M.C.); anavieira@hcrp.usp.br (A.C.d.J.V.); cacampos@hcrp.usp.br (C.C.M.); pslaurindo@hcrp.usp.br (P.d.S.L.); dimas@fmrp.usp.br (D.T.C.); bpsimoes@gmail.com (B.P.S.); 3 Department of Pediatrics, Ribeirão Preto Medical School, University of São Paulo, Ribeirão Preto 14040-900, SP, Brazil; darrigo.jr@gmail.com; 4 Cellular Thearpy Program, Oncolcínicas, São Paulo 13571-410, SP, Brazil; renatolgc@gmail.com; 5 Center for Viral Surveillance and Serological Evaluation (CeVIVAs), Butantan Institute, São Paulo 05585-000, SP, Brazil; sandra.coccuzzo@butantan.gov.br (S.C.S.); carolina.eliassabbaga@butantan.gov.br (M.C.E.); 6 Department of Sciences and Technologies for Sustainable Development and One Health, Università Campus Bio-Medico di Roma, 00128 Rome, Italy; giovanetti.marta@gmail.com; 7 Rene Rachou Institute, Oswaldo Cruz Foundation, Belo Horizonte 30190-002, MG, Brazil

**Keywords:** hematopoietic stem cell transplantation, HSCT, viral metagenomics, torque teno virus, TTV, infection, commensal viruses

## Abstract

Viral infections are one of the most important causes of morbidity and mortality among patients undergoing allogeneic hematopoietic stem cell transplantation (HSCT). Immunosuppression may lead to the reactivation of latent viruses or the acquisition of new infections, resulting in severe clinical outcomes. The early detection of viral reactivations is crucial for effective patient management and post-transplant care. In this study, we employed next-generation metagenomics to assess changes in viral abundance and detect clinically significant viruses in allogeneic HSCT patients. A total of 20 patients from the Transplant Unit of the University Hospital of the Faculty of Medicine of Ribeirão Preto, University of São Paulo were included, with plasma samples collected at three time points: D + 0 (pre-transplantation), D + 30 (30 days post-transplantation), and D + 100 (~100 days post-transplantation). A higher presence of clinically relevant viruses, such as the cytomegalovirus (CMV), the Epstein-Barr virus (EBV) and adenoviruses, were predominantly detected at D + 30. The diversity of commensal viruses, primarily anelloviruses, increased gradually, with the highest abundance and variability detected at D + 100. Viruses with clinical importance for HSCT, including CMV, adenovirus and EBV, were confirmed and characterized at the molecular level, showing generally high cycle threshold values. Our findings demonstrate a rise in anellovirus abundance following allogeneic HSCT, with the highest levels observed at D + 100. Notably, D + 30 was identified as a critical time point for the reactivation of clinically significant viruses. This study underscores the potential of metagenomics in the identification of clinically relevant viruses and highlights the importance of monitoring latent viruses in immunocompromised populations, including allogeneic HSCT patients.

## 1. Introduction

Viral infections are among some of the most significant contributors to morbidity and mortality following allogeneic hematopoietic stem cell transplantation (HSCT). These infections frequently present as opportunistic reactivations of latent viruses, primarily herpesviruses [1,2], but community-acquired respiratory infections may also play a pivotal role [3]. The risk of infection or reactivation largely depends on the degree and duration of immune suppression, with multiple factors involved, including the intensity of the conditioning regimen, donor age and immunological status, graft source, prophylaxis, graft-versus-host disease (GVHD) prophylaxis and treatment [4,5]. In general, severe infections are more common in cases of unrelated and mismatched donors, particularly in haploidentical HSCT [6].

One of the most significant infectious complications in allogeneic HSCT is the reactivation of latent herpesviruses. These viruses latently infect the hematopoietic stem cells of both the donor and recipient, and the infection can be transmitted through the transplant [7]. Clinically important herpesviruses include the cytomegalovirus (CMV), human herpesvirus-6 (HHV-6), the Epstein-Barr virus (EBV), herpes simplex viruses (HSV-1 and HSV-2), and the varicella-zoster virus (VZV). While their reactivation is usually asymptomatic in healthy individuals, in patients with compromised immune function, particularly those who have undergone HSCT, their reactivation can lead to severe systemic infections that may result in fatal outcomes. One of the most significant complications in HSCT patients are caused by CMV in the form of CMV pneumonia and enteritis, with lethality rates that can exceed 50%. EBV may be related to lymphoproliferative disorders, mainly due to T-cell depletion related to high mortality indexes. In general, the reactivation of herpesviruses includes a variety of symptoms including skin rash, fever, interstitial pneumonia, bone marrow suppression, encephalitis and GVHD [8,9,10]. Managing herpesvirus infections in allogeneic HSCT presents significant challenges. Implementing preemptive treatment, combined with the meticulous monitoring of immunosuppression, is essential for improving survival rates and minimizing the risk of permanent complications [11].

In recent years, attention has been also directed to the importance of commensal viruses in patients with immune suppression, including those undergoing allogeneic HSCT. These viruses mostly include human anelloviruses and the human pegivirus-1 (HPgV-1), both widely distributed in the human population [12,13]. It is important to note that they are not considered pathogenic, as no clinical implications have been attributed to them so far. However, the expression level and viral load of anelloviruses have been directly correlated with the degree of immune suppression. Monitoring anellovirus, and especially Torque Teno Virus (TTV), DNA levels in the blood can be regarded as a surrogate tool for identifying patients at risk of infectious complications, consequences of immune suppression, and for adjusting immunosuppressive therapy [14,15,16]. On the other hand, HPgV-1 has been implicated in the exertion of beneficial effects in HIV-infected patients, as shown by the higher CD4+ T-cell counts and lower HIV viral loads, thus possibly delaying the progression to AIDS [17,18,19,20]. The precise mechanisms through which HPgV-1 may influence HIV infection remain poorly understood. However, it has been suggested that HPgV-1 may directly interfere with HIV replication, possibly through the HPgV-1 E2 protein, which may participate in the inhibition of HIV entry into CD4+ cells. Another potential explanation involves the ability of HPgV-1 to alter the cytokine profile of co-infected individuals, prompting peripheral blood mononuclear cells to produce higher levels of interferon-gamma in co-infected patients compared to those without HPgV-1 infection [19]. In the context of patients undergoing allogeneic HSCT, it is unclear whether HPgV-1 exerts any significant effects. Observations indicate that patients with allogeneic HSCT who are infected with HPgV-1 exhibit impaired natural killer cell immune reconstitution but not T cell reconstitution compared to HPgV-1-negative patients. This finding suggests a potential association between the presence of HPgV-1 and post-transplantation immune reconstitution [12]. Additionally, the monitoring of HPgV-1 RNA levels before or after transplantation may identify patients with an increased risk of opportunistic infections or GVHD [13].

In this study, we performed next-generation sequencing and metagenomic analyses of plasma samples obtained at three different time points from patients who underwent HSCT. We analyzed the virome patterns before transplantation (D + 0), 30 (D + 30), and 100 (D + 100) days after the transplantation. We evaluated the composition, dynamics, and viral changes over this period, with a particular focus on commensal viruses and clinically significant ones. Clinically relevant viruses were confirmed in individual clinical samples, and their molecular characteristics were evaluated.

## 2. Materials and Methods

### 2.1. Study Population and Study Design

In this study, we performed viral metagenomics in 20 allogeneic HSCT transplant recipients from the Hematology Department, Clinical Hospital of Ribeirão Preto, Faculty of Medicine of Ribeirão Preto. We collected ~4 mL of blood from all patients prospectively at three time points for the comparison of viral abundance (i) before the start of the conditioning regimen (D + 0); (ii) 30 days after the transplantation D + 30 and (iii) ~100 days after the transplantation (D + 100). The number of samples obtained from these patients totalized 49 samples (D + 0: 20 samples, D + 30: 18 plasma samples and D + 100: 11 samples). Two patients died before D + 30, five patients died before D + 100 and one patient could not be assessed for D + 100 sampling. The information on the pre-transplantation serological status, with regards to CMV and EBV infections, was obtained from the patients’ records. The Institutional Ethics Committee of the University Hospital of Ribeirão Preto and University of São Paulo approved this study under the following process number: CAAE 05142818.7.0000.5440.

### 2.2. Next-Generation Sequencing

Initially, 600 μL of patient plasma were pre-treated with the Turbo DNase (Turbo DNA-free kit, Thermo Fisher Scientific, Waltham, MA, USA) to remove free host and bacterial DNA, with DNase incubation at 37 °C for 30 min. Following DNase inactivation, five individual samples were pooled and the total pooled volume was extracted using the High Pure Viral Nucleic Acid Large Volume Kit (Roche, Basel, Switzerland) according to the manufacturer’s instructions. The only variations were the use of the GenElute™ LPA Carrier (Merck, Darmstadt, Germany) for nucleic acid concentration and the use of isopropyl alcohol for nucleic acid precipitation. The pooling strategy was based on the collection dates (D + 0, D + 30, and D + 100) to compare and evaluate viral abundance. A total of 8 pools were prepared, each containing 5 to 8 plasma samples. Pooling clinical samples was implemented to reduce sequencing costs while simultaneously enabling the inclusion of a larger number of patients in the study. After extraction, viral nucleic acids underwent reverse transcription using the Superscript III First-Strand Synthesis System (Thermo Fisher Scientific, Waltham, MA, USA) following the manufacturer´s instructions. The amplification of cDNA was performed using the QuantiTect Whole Transcriptome Kit (QIAGEN, Hilden, Germany). Genomic libraries for sequencing were prepared using the Illumina DNA Prep Kit (Illumina, San Diego, CA, USA) and the IDTR for Illumina R DNA/RNA UD Indexes Set plate B, following the manufacturer’s instructions. The library quality was assessed using a BioAnalyzer 2100 (2100 Expert Software, Agilent Biotechnologies, Santa Clara, CA, USA) with a High Sensitivity DNA Chip (Agilent Biotechnologies, Santa Clara, CA, USA). The sequencing of the dual-indexed libraries was performed on an Illumina NovaSeq 6000 platform, using the NovaSeq 6000 S1 Reagent Kit v. 1.5, 300 cycles (Illumina, San Diego, CA, USA), in accordance with the manufacturer’s instructions.

### 2.3. Bioinformatic Analysis

To access the abundance of virome in the plasma samples of transplant recipients, we employed an in-house optimized bioinformatics pipeline. The obtained raw sequence data were initially subjected to quality control using FastQC v.0.11.08 [20]. To ensure high-quality reads, we trimmed and filtered low-quality reads and adapters using Trimmomatic v.0.39 [21] and Cutadapt v. 2.4 [22]. Only reads with a Phred score > 30 were retained for further bioinformatics analysis. Additionally, we conducted automatic trimming and quality control to enhance the overall quality of the reads using AfterQC software v. 0.9.7 [23]. Subsequently, for taxonomic classification, we used the Kraken v.2.0.8 program [24] with the Nucleotide Whole Genome Shotgun and the Nucleotide GenBank databases, both from NCBI. To map the obtained reads against reference genomes, we used the BWA v.0.7.17 software [25]. For the generation of larger contigs and scaffolds, we used SPAdes 3.13.0 [26]. Finally, to classify the formed contigs we used the Diamond 0.9.29 software [27].

### 2.4. Direct Confirmation of Viruses with Clinical Importance

Viral nucleic acids were manually extracted from plasma using the QIAamp Viral RNA Mini Kit (QIAGEN, Hilden, Germany) according to the manufacturer’s instructions. Based on the initial assessment of viral abundance obtained in the metagenomic analysis, we identified the presence of clinically significant viruses in HSCT and prioritized their detection using molecular assays. For all identified viruses with clinical importance (HCMV, adenovirus type 7 and EBV), we employed TaqMan^®^ real-time PCR for the detection and evaluation of the cycle threshold. The primers and probes used in the molecular reactions are detailed in Table 1. For real-time PCR detection, we used a concentration of 400 nM for each primer and 250 nM for each probe, in a final reaction volume of 25 µL, supplemented with 1X TaqMan™ Universal PCR Master Mix (Thermo Fisher Scientific, Waltham, MA, USA). Amplification was carried out on a 7500 real-time PCR system (Thermo Fisher Scientific, Waltham, MA, USA) under the following conditions: an initial step of 50 °C for 5 min, followed by denaturation at 95 °C for 5 min, and 40 cycles of 95 °C for 15 s and 60 °C for 1 min. The presence of adenoviruses in plasma was further confirmed by nested PCR using consensus primers that detect the majority of adenovirus subtypes [28].

## 3. Results

### 3.1. Demographic and Clinical Data of the Tested Patients

Of the study group, 40% of participants were female (n = 8) and 60% were male (n = 12), with a median age of 38.85 years (range 18–66; ±15.5). Among the included patients, five (25%) developed acute graft-versus-host disease (GVHD) affecting the skin, liver, or gastrointestinal system, all of whom were treated systemically with corticosteroids. The demographic and clinical characteristics of the tested patients are summarized in Table 2.

### 3.2. Viral Abundance in Allogeneic HSCT

**Figure 1:**
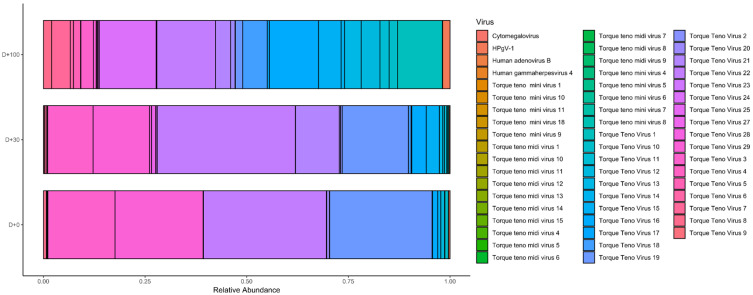
Horizontal barplot of the relative abundance of the virome of transplanted patients at three collection time points called D + 0, D + 30, and D + 100. It is possible to observe a large amount of TTV at all three collection points. As time goes by, the anelloviruses become more diverse. On D + 30, EBV and CMV, as well as human adenovirus type B, readings begin to be identified, indicating that they tend to reactivate. By D + 100, we observe a higher abundance of viruses; the total reads of TTV and HPgV-1 increases.

The virome of HSCT patients was evaluated at three time points: D + 0, D + 30, and D + 100. We observed variations in both virome abundance and diversity across these time points, with the highest viral richness occurring at D + 100 after transplantation. During this period, viral abundance was particularly high, with a notable diversity of anelloviruses. The most prevalent species across all pools were the commensal viruses TTV 1, 16, 18, 22, and 24, with an average of 655,148 reads per pool. At D + 30, we observed the presence of herpesviruses (CMV, EBV), which tend to reactivate following allogeneic HSCT, though their read numbers were relatively low. Additionally, at D + 30, the human adenovirus type 7 was detected in one pool and was subsequently confirmed in an individual sample. At D + 0, we observed a high abundance of various anelloviruses and a significant number of CMV reads in one pool, which was subsequently confirmed in the individual sample with low cycle threshold value. Herpesvirus diversity at this stage was lower. At D + 100, viral abundance was again predominantly composed of anelloviruses, with several herpesviruses (EBV, CMV) present in low read numbers. Virus abundance across collection periods is depicted in Figure 1.

**Figure 2:**
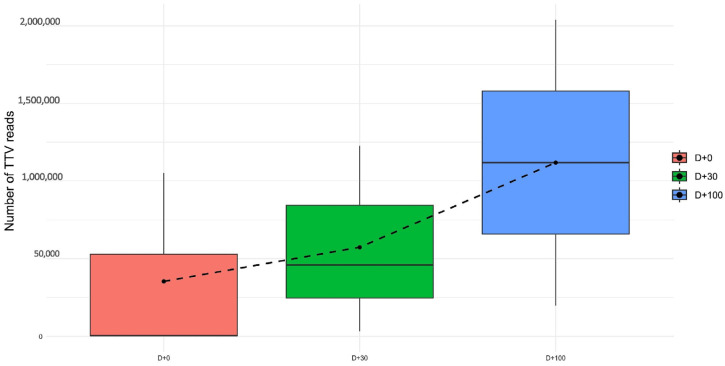
Boxplot of the number of TTV (Torque Teno Virus) reads on different collection days (D + 0, D + 30 and D + 100). The dots represent the mean number of sequence reads, and the lines show how they vary over time. An increase in the mean number of TTV readings was observed over time, with D + 100 presenting the highest mean number of readings (1,118,458, IQR [658,347; 1,578,570]). Dashed line: evolution of the total numbers of TTV reads over the period of collection.

We evaluated the variation in TTV read counts across the different collection days. The results indicated a gradual increase in total TTV reads over time. At D + 0, the mean TTV reads were 353,204 (IQR [4028; 528,109]) Thirty days after the transplantation, the rate of TTV reads increased, with a mean value of 573,121 (IQR [246,404; 842,979]). By D + 100, the highest mean TTV reads were observed, with 1,118,458 IQR [658,347; 1,578,570]) reads per pool. Although this observed increase suggests a trend toward significance, the Kruskal–Wallis test did not yield significant results (*p*-value = 0.3679), likely due to the limited number of pooled samples available for analysis. Nonetheless, this rise may indicate a dynamic change in TTV levels over time. The trend is visually represented in the boxplot, which highlights the differences in read distributions across the sampling time points, underscoring the importance of monitoring TTV levels in HSCT patients (Figure 2).

**Figure 3:**
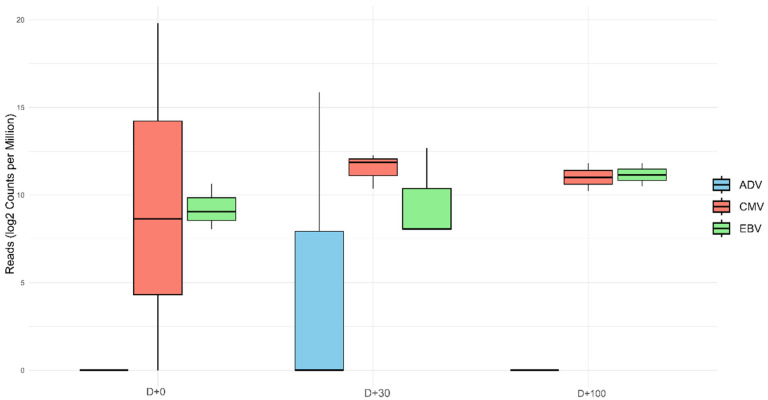
Box plot depicting the total abundance of CMV, EBV and ADV reads across different collection points (D + 0, D + 30, and D + 100) to illustrate their variation over time. At D + 0, the highest abundance of CMV reads (median = 28, IQR [19.00; 32.00]) was observed; this was due to the low CMV Ct value in one of the samples (Ct = EBV was also identified (median = 4, IQR [3.00; 8.00]). At D + 30, the three viruses were identified. At D + 100, ADV was no longer identified in any pool; we could still identify CMV and EBV at this time point. Reads were normalized using log2 counts per million reads.

We also evaluated the variation of read counts of CMV, EBV and adenoviruses along the different collection points (Figure 3). EBV and CMV reads were identified at all collection points, while adenovirus reads were identified only at D + 30. For EBV, on D + 100, the median read count was notably higher at 19 (IQR [15.00, 23.00]). In comparison, D + 30 showed a slightly lower median of 2 (IQR [2.00, 26.00]), while D + 0 had a median of 4 (IQR [3.00, 8.00]). For CMV, on D + 0, a significant outlier was observed, with a high number of reads that were due to the low cycle threshold detected in one patient (see Table 3, patient RP18). A total of 6882 CMV reads were detected in these sample pools. This resulted in a high variance in the boxplot, reflected by a median of 3 (IQR [1.00, 3442.00]). By D + 30, the median increased substantially to 28 (IQR [19.00, 32.00]).

At D + 100, a slight decrease was observed, with the median dropping to 18 (IQR [13.00, 22.00]). The adenovirus type 7 was detected exclusively at D + 30, in one pool, with a total of 448 reads. This infection was consequently confirmed by real-time PCR. This value significantly influenced the distribution for that day, resulting in a median of 149 (IQR [0.00, 224.00]). The trends of the read distribution of CMV, EBV and adenovirus type 7 are shown in Figure 3.

### 3.3. Molecular Prevalence of the Tested Viruses and CMV/EBV Serological Pre-Transplantation Status

CMV presence was confirmed by real-time PCR in seven HSCT patients (n = 7/20; 35%). Among these, samples were collected at three time points, for five patients, while for two patients (RP3 and RP19), plasma samples were only obtained at D + 0 and D + 30 due to the fatal outcome of the transplantation. In most cases, CMV DNA was detected at D + 30 (in four patients) with a high mean Ct value of 38.0 (range 35.6–39.1). In one patient (RP18), CMV was detected at D + 0, with a relatively low Ct value (26.7). CMV was detected at D + 100 in two patients (RP9 and RP10) with a high mean Ct value of 38.8. CMV was detected only once in each patient along the collection points. The characteristics of CMV infection in the positive patients are detailed in Table 3. The human adenovirus type 7 was confirmed as a co-infection with CMV in the plasma of one patient (RP19) at D + 30, with a Ct value of 31.2. This patient was a 66-year-old male who underwent haploidentical HSCT with a diagnosis of acute myeloid leukemia. Additionally, EBV was confirmed in one patient at D + 100 with a high Ct value of 38.3. This patient was a 60-year-old female who underwent related allogeneic transplantation with a primary diagnosis of severe aplastic anemia. The amplification data for the tested viruses can be found in Table 3. We also evaluated the pre-transplant serological status (IgM and IgG) for CMV and EBV in all patients who tested positive for these agents using real-time PCR in one of the collections. All patients were positive for CMV and EBV IgG prior to transplantation, and none tested positive for CMV or EBV IgM after, indicating no reactivation. The serological data for CMV and EBV in patients with positive real-time PCR results are presented in Table 4.

## 4. Discussion

Viral infections pose a significant risk of morbidity and mortality in patients undergoing HSCT. Given the limited availability of effective antiviral therapies, the management of these infections plays a crucial role in reducing lethality. Identifying the critical periods post-transplantation, when latent viruses are most likely to reactivate, is essential for shaping optimal preventive strategies. Such insights can further mitigate the risk of infectious complications and guide clinical interventions aimed at improving patient survival outcomes. In this study, we applied viral metagenomics to examine the changes in viral composition and the presence of clinically significant viruses in the context of allogeneic HSCT within a cohort of 20 patients. Plasma samples were collected prospectively at different time points to identify critical periods for the presence of viral infections or reactivations and changes of the total viral abundance over this period, including that of the clinically important viruses.

The observed viral abundance showed that the most prevalent viruses identified by metagenomics were anelloviruses, which were represented as a high diversity of alpha-, beta-, and gamma-anelloviruses. Chronic anellovirus infections represent a common finding among allogeneic HSCT patients. A high prevalence ranging between 97% and 100% has been documented [30,31]. Furthermore, the anelloviruses, generally in the form of multiple co-infections, may account for up to 93% of the total viral abundance [32,33]. We observed an accentuated increase in anellovirus abundance and diversity, with the highest values recorded at day 100 (D + 100) post-transplantation. Other studies have also shown that TTV levels peak at D + 100 post-transplantation and might be associated with poorer clinical outcomes [33,34]. To date, the clinical significance of anelloviruses remains uncertain. Due to their widespread distribution in the human population, anelloviruses are not considered pathogenic [32]. However, in the context of allogeneic HSCT, both viral loads and anellovirus abundance may increase. Consequently, anelloviruses have been increasingly recognized as important indicators of immune suppression in allogeneic HSCT patients [35]. Therefore, quantifying the viral load of these viruses has been suggested as a surrogate biomarker for assessing levels of immune competence and reconstitution [36], including in HSCT patients [35].

Metagenomic analysis also identified the presence of clinically significant viruses, and fluctuations in their read numbers across different collection points were examined. CMV was detected at all collection points, with a notably high read count at D + 0, attributed to the presence of a sample with a low cycle threshold in one of the pools. At all other collection points, CMV exhibited low read counts, consistent with the very low cycle thresholds observed in individual positive samples. In contrast, EBV reads were detected in low numbers, likely reflecting a very low viral load. EBV infection was confirmed in only one individual case at D + 100, again with a very low cycle threshold. Similarly, the adenovirus type 7 was identified exclusively at D + 30 in a single pool, leading to the subsequent confirmation of infection in the plasma of one patient.

The detection of clinically significant infections—CMV, EBV, and adenovirus type 7—in the tested pools necessitated a more thorough investigation of the molecular characteristics of these infections. This included confirmation in the individual samples comprising each pool through molecular methods and an evaluation of their cycle thresholds. Direct confirmation was performed on these samples to gather information on the dynamics of these infections and to rule out contamination or bioinformatics errors. At day 30 (D + 30), we observed the highest CMV prevalence across and CMV infection was confirmed by real-time PCR in a total of seven allogeneic HSCT patients (one at D + 0, four at D + 30, and two at D + 100). With the exception of one patient (D + 0), all cases of CMV infection were characterized by high cycle threshold (Ct) values, suggesting a low viral load (mean Ct ~38.0). The highest CMV prevalence was observed at D + 30, which also coincided with the detection of other clinically important pathogens, such as adenoviruses. Along with the high CMV prevalence at D + 30, we were able to confirm other important for HSCT transplantation viruses, like the human adenovirus type 7, in this period. In this study, the patients did not receive CMV prophylaxis but were monitored weekly for CMV infection until D + 100 and promptly received preemptive treatment if CMV reactivation was registered. This meticulous protocol might explain the observed low CMV cycle thresholds. CMV infection is one of the most significant complications in allogeneic HSCT, remaining a major factor in morbidity and mortality, with reactivation peaks typically occurring not only at D + 30 but also potentially extending to D + 100 post-transplantation [37,38]. This pattern was similarly observed in our investigation, where CMV reactivation occurred at higher rates at D + 30 and also at D + 100. Nonetheless, the lowest CMV cycle threshold was observed prior to transplantation, possibly as a consequence of the induction therapy, despite the absence of CMV disease (CMV IgM negative). Due to precise CMV load monitoring following transplantation and the prompt initiation of antiviral therapy, no significant increase in CMV load was observed in the post-transplantation period. An additional factor contributing to the observed CMV reactivation rates may be the positive serological status present in all patients confirmed to be positive by CMV PCR. Without prophylaxis and the monitoring of CMV reactivation after allogeneic HSCT, nearly 80% of transplanted patients experience CMV reactivation. Therefore, careful monitoring of CMV load following allogeneic HSCT, particularly in seropositive recipients, is crucial for the timely initiation of anti-CMV treatment, thereby preventing severe CMV disease [1].

Another clinically significant virus detected in allogeneic HSCT patients through metagenomics and subsequently confirmed by real-time PCR in plasma was the adenovirus type B (type 7). Following primary infection, adenoviruses may persist in various tissues, including adenoidal and tonsillar T-lymphocytes, from which reactivation of the latent virus can occur, particularly in cases of immunosuppression, as seen in patients undergoing allogeneic HSCT. Additionally, these viruses may be community-acquired [39]. Adenoviruses are associated with significant morbidity and mortality among transplant recipients, with clinical manifestations ranging from asymptomatic viremia to respiratory and gastrointestinal diseases, hemorrhagic cystitis, enteritis, nephritis, hepatitis, pneumonia, encephalitis, and severe disseminated illness with multiorgan involvement [40,41]. Although adenovirus disease is more frequently diagnosed in pediatric HSCT patients, in our case, the infection was identified in an adult patient with a concurrent CMV infection who died before D + 100 post-transplantation. Consequently, we could not observe the adenovirus dynamics. However, risk factors for adenovirus reactivation or infection, including haploidentical transplantation, were noted [42]. Adenovirus infection was detected around day +30 post-transplantation, aligning with the existing literature, which indicates that adenovirus detection typically occurs before D + 100 post-transplantation [43].

Finally, at D + 100, EBV was detected at high Ct in one patient with a previous CMV infection at D + 0. Like the other herpesvirus representatives, EBV may reactivate during immune suppression, including in allogeneic HSCT patients [44]. The reactivation of EBV after allogeneic HSCT might be associated with clinical complications that include the development of post-transplant lymphoproliferative disorder (PTLD). PLTD is associated with a loss of the cytotoxic T-cell control and unchecked B-cell activation, with incidence rates between 1 and 10% among allogeneic HSCT. The monitoring of the EBV levels in peripheral blood is used to predict the likelihood of PLTD development. A reduction in immune suppression and preemptive treatment with rituximab is warranted upon a stable increase in the viral load [45].

This study is subject to several limitations. First, the tested sample size was relatively small, which may limit the generalizability of our findings. Additionally, the cohort was composed of patients with a wide range of clinical conditions, varying types of transplantation, and diverse treatment regimens. This heterogeneity introduces potential confounding factors and limits the ability to draw conclusions about specific subgroups. Another important limitation is related to the methodology of viral metagenomics. Due to financial constraints, we employed plasma pooling for the analysis. While this approach was cost-effective, it precluded us from obtaining individualized data on the presence and abundance of commensal viruses. As a result, variations in viral loads across individual patients were not captured, which may affect the precision of our virome characterization. Future studies would benefit from the inclusion of individual-level viral metagenomic profiling to better understand the variability of viral populations across distinct clinical cases.

## 5. Conclusions

In conclusion, this study provides a comprehensive analysis of viral dynamics at critical time points in patients undergoing allogeneic HSCT. Our findings demonstrate a progressive increase in total anellovirus abundance, peaking around day 100 (D + 100) post-transplantation. This probably suggests that shifts in the abundance of the metavirome may serve as indirect indicators of immune status in allogeneic HSCT patients. Real-time PCR analyses of individual samples revealed a higher frequency of tested viruses at day 30 (D + 30), highlighting this time point as a critical period for viral monitoring. Additionally, our study offers valuable insights into viral presence during critical post-transplantation periods, which could inform improvements in current methods for viral detection and surveillance in this high-risk population, ultimately contributing to more effective patient management and post-transplantation care.

## Figures and Tables

**Table 1:** Primers and probes used for molecular screening.

Virus	Primers (5′-3′)	Probe FAM-MGB (5′-3′)	Genomic Localization *
Cytomegalovirus (CMV)	Forward: ACCGTCTGCGCGAATGTTA Reverse: TCGCAGATGAGCAGCTTCTG	CACCCTGCTTTCCGAC	FP 143,285–143,303 RP 143,332–143,351 Probe 143,305–143,320 (KU221100)
Epstein-Barr virus (EBV) [29]	Forward: TCAACCTCTTCCATGTCACTGAGA Reverse: TGGGTGAGCGGAGGTTAGTAA	TCAGCCCCTCCACCAGTGACAATTC	FP 77,792–77,812 RP 77,876–77,899 Probe 77,829–77,853 (MK973061)
Adenovirus type 7 (in-house designed)	Forward: CGCCGGACAGGATGCTT Reverse: CTACGGTCGGTGGTCAC	AGTCCGGGTCTGGTGCAGTTCGCC	FP 18,438–18,454 RP 18,559–18,757 Probe 18,466–18,489 (OP270254)

* Genomic localization based on the reference genome.

**Table 2:** Demographic and clinical parameters of the tested patients.

Demographic and Clinical Parameters	Frequency
***Gender***	
Male	n = 12/60%
Female	n = 8/40%
***Age***	
Mean	38.85
Range	18–66 years
***Clinical condition***	
Sickle cell disease	n = 1/5%
Chronic myeloid leukemia	n = 3/25%
Acute lymphoblastic leukemia	n = 5/25%
Acute myeloid leukemia	n = 5/25%
Aplastic anemia	n = 6/30%
***Type of transplant***	
Haploidentical	n = 6/30%
HLA identical	n = 11/55%
Unrelated donor	n = 3/25%

**Table 3:** Molecular characteristics of the viruses detected in plasma among patients with HSCT.

Patient ID	D + 0	D + 30	D + 100
RP3	-	CMV (Ct = 39.1)	-
RP5	-	CMV (Ct = 38.7)	Death
RP9	-	-	CMV (Ct = 38.7)
RP10	-	-	CMV (Ct = 38.8)
RP18	CMV (Ct = 26.7)	-	EBV (Ct = 38.3)
RP19	-	CMV (Ct = 38.7), Adenovirus B (Ct = 31.2)	Death
RP21	-	CMV (Ct = 35.6)	-

**Table 4:** Serological pre-transplantation status for CMV and EBV in the PCR positive patients.

ID	Date of Testing	Date of Transplantation	CMV IgG	CMV IgM	EBV IgG	EBV IgM
RP3	26 September 2018	17 May 2019	+	−	+	−
RP5	3 October 2018	4 July 2019	+	−	+	−
RP9	16 July 2019	31 October 2019	+	−	+	−
RP10	5 November 2019	22 November 2019	+	−	+	−
RP18	17 December 2018	3 September 2020	+	−	+	−
RP19	6 August 2020	23 October 2020	+	−	+	−
RP21	31 July 2020	29 October 2020	+	−	+	−

## Data Availability

Data are contained in the article.
